# 基于ctDNA的MRD检测在早期非小细胞肺癌根治术后的应用价值

**DOI:** 10.3779/j.issn.1009-3419.2021.102.44

**Published:** 2021-12-20

**Authors:** 世华 窦, 鸿生 谢, 林 杨

**Affiliations:** 518020 深圳，暨南大学第二临床医学院（深圳市人民医院）胸外科 The Second Clinical Medical College, Jinan University (Shenzhen People's Hospital), Department of Thoracic Surgery, Shenzhen 518020, China

**Keywords:** 肺肿瘤, ctDNA, MRD检测, 辅助治疗, 根治术, Lung neoplasms, ctDNA, MRD detection, Adjuvant therapy, Radical surgery

## Abstract

肺癌是世界上最常见的恶性肿瘤，其中非小细胞肺癌（non-small cell lung cancer, NSCLC）约占肺癌总数的85%。接受手术的NSCLC患者的5年总生存期（overall survival, OS）从Ⅰa1期的92%到Ⅲb期的26%，持续下降的生存期使其临床上有强烈的精准辅助治疗需求，以根除分子残留病灶（molecular residual disease, MRD）。目前循环肿瘤DNA（circulating tumor DNA, ctDNA）作为提示MRD的分子指标逐渐从实验室走向临床。最新共识提出在围手术期NSCLC患者外周血中可稳定检测出丰度≥0.02%的ctDNA，是基于ctDNA作为MRD指标的可能。MRD检测技术支持了NSCLC根治术后进行监测的可能性，而且ctDNA能比NSCLC治疗后的影像监测更早地预示着疾病的复发，为精准辅助治疗方案的制定提供了宝贵依据。在早期NSCLC术后辅助治疗研究中，不同指南对是否应该进行适合的辅助治疗存在分歧，而MRD可以作为一个更精准的预测指标，对术后辅助治疗进行指导，使患者能在疾病治疗中获益。

## Abstract (en)

Lung cancer is the most common malignant tumor in the world, among which non-small cell lung cancer (NSCLC) accounts for about 85% of the total number of lung cancers. The 5-year overall survial (OS) of radical surgery NSCLC patients ranged from 92% in stage Ⅰa1 to 26% in stage Ⅲb, and the continuously decreasing survival time made it a strong clinical need for precise adjuvant therapy to eradicate molecular residual disease (MRD). At present, circulating tumor DNA (ctDNA) as a molecular indicator of MRD has gradually moved from the laboratory to the clinic. The latest consensus proposes that ctDNA with abundance ≥0.02% can be stably detected in the peripheral blood of perioperative NSCLC patients, which is based on the possibility of ctDNA as an MRD indicator. MRD detection technology supports the possibility of monitoring after radical treatment of NSCLC, and ctDNA can predict the recurrence of the disease earlier than the imaging monitoring after treatment of NSCLC, providing valuable time for timely adjustment of adjuvant therapy. In the studies on early postoperative adjuvant therapy of NSCLC, different guidelines differ on whether appropriate adjuvant therapy should be carried out, while MRD can be used as a more accurate predictor to guide postoperative adjuvant therapy, so that patients can benefit from the disease treatment.

根据世界卫生组织国际癌症研究机构（International Agency for Research on Cancer, IARC）发布的2020年全球最新癌症数据，肺癌估计有220万新发和180万死亡病例^[1]^。其中，非小细胞肺癌（non-small cell lung cancer, NSCLC）约占肺癌总数的85%^[2]^。对于早期术后患者，推荐临床访视和X线计算机断层扫描（computed tomography, CT）作为肿瘤监测的主要手段。虽然CT扫描可以检测到术后患者的疾病进展早于X线，但两组患者的总体生存率没有统计学差异^[3]^，表明传统的放射学方法在术后随访中可能已经达到极限。如何识别根治性手术后的分子残留病灶（molecular residual disease, MRD）、预测疾病复发或转移和指导术后治疗，这仍然是一系列亟需解决的问题。本文将综述循环肿瘤DNA（circulating tumor DNA, ctDNA）与MRD的关系、ctDNA的检测方法、ctDNA的应用，以及通过MRD的检测结果指导术后辅助治疗可产生显著的临床相关性。

## 1 ctDNA与MRD的关系

ctDNA是血液中肿瘤衍生的片段化DNA，ctDNA直接来自肿瘤或循环肿瘤细胞，主要由单链或双链DNA以及单链与双链DNA的混合物组成，存在于血浆或血清中^[4]^。ctDNA中常包含突变、缺失、插入、重排、拷贝数异常以及甲基化等相关基因突变信息^[5]^。目前以ctDNA作为MRD的指标最常见^[6]^，对晚期肺癌的研究^[7-12]^表明，ctDNA分析在肿瘤基因突变谱检测、疾病进展监测、评判肿瘤负荷和预测药物疗效等方面具有重要的临床价值。在肠癌、乳腺癌、肌层浸润性尿路上皮癌及肺癌等术后患者中，基于术后血浆ctDNA发现MRD，相较与影像学显示出对肿瘤复发及转移预测的明显优势^[13-17]^。在2021年第18届中国肺癌高峰论坛上对肺癌MRD达成了专家共识：肺癌MRD指的是经过治疗后，传统影像学（包括正电子发射体层显像/CT（positron emission tomography/CT, PET/CT）或实验室方法不能发现，但通过液体活检发现的肿瘤来源分子异常，代表着肺癌的持续存在和临床进展可能。肺癌分子残留病灶：指的是在外周血可稳定检测出丰度≥0.02%的ctDNA，包括肺癌驱动基因或其他的Ⅰ类/Ⅱ类基因变异^[18]^。经过治疗后仍能测出丰度≥0.02%的ctDNA，表明存在MRD。

## 2 ctDNA MRD常用的检测方法

DNA测序技术的进步和我们对肿瘤分子生物学的理解，通过检测术后MRD识别疾病复发的高风险患者，并在辅助治疗环境下调整临床方案，以优化风险分层，这一目标是可以用现有技术实现的^[19]^。

基因测序的主要策略为检测肿瘤特异性突变或预定义的基因组区域（靶向ctDNA策略）。此类型分析基于非常灵敏的技术使用，一类是PCR的分析，如二代的实时荧光定量PCR（real-time quantitative PCR, qPCR）、突变扩增阻滞系统（amplification refractory mutation system, ARMS）和三代的微滴数字PCR（droplet digital PCR, DdPCR）；PCR技术在ctDNA检测中从二代到三代的迭代完成了特异性和灵敏性提升（灵敏度从0.1%到0.001%^[20-22]^），但因不能全面检测驱动变异之外的突变，这限制了它在多重检测中的应用。另一类是基于二代测序（next generation sequencing, NGS）的方法，NGS检测ctDNA技术路线有两大方向，一个是基于tumor-naïve的路线，代表研究有Lung-CLiP研究^[23]^，研究者采用肿瘤个体化分析深度测序法（cancer personalized profiling by deep sequencing, CAPP-Seq），其平均测序深度达23, 570×，将检出的分子突变信息结合基因组捕获区域的基因拷贝数变异（copy number variations, CNV）等信息，纳入评估模型。但在整体98%特异性下，Ⅰ期、Ⅱ期、Ⅲ期NSCLC敏感性仅为41%、54%、67%；另一个是基于tumor-informed的路线，代表方法为个体化定制监控方案（personalized cancer monitoring, PCM），Zviran等^[24]^以原发肿瘤突变谱为基础，利用全基因组测序（whole genome sequencing, WGS）对整个基因组进行检测（MRDetect技术），通过观测整个基因组寻找肿瘤组织已经存在的突变，应用极大的广筛ctDNA测序代替加深深度测序来提高敏感性。基于此，Signatare首先使用全外显子测序（whole exome sequencing, WES）的方法，筛选出肿瘤组织携带的变异，然后针对这些突变定制患者专属的基于扩增的NGS panel，通过超高深度100, 000×的深度测序，最大化地对每个患者特有的肿瘤变异进行个体化跟踪。另一家公司ArcherDX在这个基础上，在单点测序上增加了单分子标签技术（unique molecular identifiers, UMI），又进一步提升了单点检测的敏感性。Signatare和ArcherDX这两种方法均可以归纳为PCM。PCM在特异性监控的同时，能达到对0.01%到0.1%级别的变异丰度较好的覆盖，基本实现早期肿瘤患者ctDNA变异的覆盖。但这一方法学仍有一定的局限性，由于肿瘤的克隆进化及二次原发肿瘤的存在，患者新原发病灶的跟踪、监测以及新克隆的出现，对于基于tumor-informed的PCM来说是无法覆盖的。例如在TRACERx研究^[16]^中，Ⅰ期采用Signatera，Ⅱ期采用ArcherDX，结果发现其中大约12%的患者复发肿瘤病灶从分子上定义是一个新原发的肿瘤，而对于这种新原发病灶来说PCM的方法无法做到覆盖和监控。而MRD的检测维度也是不断丰富，除了基于ctDNA变异的单一监控维度，ctDNA甲基化也成为MRD研究中重要的角色。

## 3 基于ctDNA的MRD检测阳性预示NSCLC术后较差的无复发生存期（relapse-free survival, RFS）与复发

在非转移性肺癌患者中，部分患者可以通过初次手术切除、放射治疗和包括化疗在内的综合治疗方法治愈^[25, 26]^。在一线治疗之后影像学只能监测到宏观疾病复发，但是由于治疗后肿瘤组织的坏死等因素，影像学检测发现复发或进展病灶时，患者的全身肿瘤负荷已明显升高。因此，NSCLC根治术后MRD检测是否能识别有复发风险的患者，并且可以在肿瘤负荷最低的情况下进行个性化辅助治疗是一个值得深入研究的课题^[17, 27]^。

Peng等^[27]^研究表明，未检测到ctDNA（ctDNA转为阴性）患者的RFS和总生存期（overall survival, OS）明显好于术后可检测到ctDNA（ctDNA仍为阳性）的患者（*P* < 0.05）。当与美国癌症分期联合委员会（American joint Committee on cancer, AJCC）分期一起使用时，ctDNA提供了一个相对精确的风险分层。Ohara等^[28]^用CAPP-seq检测ctDNA，术后ctDNA阳性提示更短的RFS（*P*=0.015）。Chen等^[29]^发现术后3 d检测ctDNA与无病生存期（disease-free survival, DFS）密切相关（*P*=0.018），基于以上，我们将有可能在术后动态监控MRD，并且这一指标具有预测肺癌复发的可能性。Chaudhuri等^[17]^的研究中，与治疗结束后未检测到ctDNA的患者相比，在治疗后任一时间点均可检测到ctDNA的患者的生存率显著降低（*P* < 0.001）（表 1）。综上所述，通过MRD检测可以更早识别有复发风险的患者，是NSCLC根治术后的一个实用预后因素。

**表 1:** ctDNA在早期NSCLC术后的应用 Application of ctDNA after early NSCLC surgery

Detect method	Preoperative ctDNA positive blood sample/ total blood sample						Postperative ctDNA positive blood sample/ total blood sample					Total number of relapses	Total number of postoperative ctDNA positive recurrences	Follow-up time (mon)
	Total	Ⅰ	Ⅱ	Ⅲ	Ⅳ		Total	Ⅰ	Ⅱ	Ⅲ	Ⅳ			
CAPP-seq^[17]^	3/5	1/1	1/1	1/3	0		0/5	0/1	0/1	0/3	0	0	0	31.5
cSMART^[27]^	46/77	18/41	13/18	13/16	2/2		30/71	11/38	7/17	10/14	2/2	35	19	46
CAPP-seq^[28]^	8/20	1/5	2/8	5/7	0		4/20	0/5	1/8	3/7	0	5	3	12
cSMART^[29]^	26/26	4/4	5/5	17/17			9/26	1/4	2/5	6/17		9	6	17.7
Postoperative ctDNA can turn negative from positive and consistently positive ctDNA predicts relapse and poor recurrence-free survival (RFS). NSCLC: non-small cell lung cancer; ctDNA: circulating tumor DNA; cSMART: cyclic single-molecule amplification and re-sequencing techniques; CAPP-seq: cancer personalized profiling by deep sequencing.														

术后ctDNA检测比影像学检查更早提示疾病进展或复发，Xia等^[8]^表示甲基化水平和最大等位基因频率（maximum allelic fraction, maxAF）的降低反映了治疗效果，而逐渐升高反映了即将到来的疾病进展（progressive disease, PD）。在使用放射成像评估PD之前，分别有6例和5例患者平均提前3.0个月和1.9个月观察到甲基化水平和maxAF升高。Peng等研究^[27]^表明19例（63.3%）患者术后ctDNA先于影像学表现或临床症状出现，平均时间为12.6个月。而用ctDNA检测肿瘤复发的中位时间比CT检测提前165 d^[29]^，Chaudhuri等^[17]^根据实体肿瘤的疗效评价标准1.1版（Response Evaluation Criteria in Solid Tumors Version 1.1, RECIST 1.1）标准也证实了其研究中72%的患者（共19例）的ctDNA检测先于CT进展，中位时间为5.2个月。综上所述，术后ctDNA检测比影像学检测更灵敏，能更早提示疾病进展或复发，有助于患者肿瘤负荷处于较低状态下进行辅助诊断，从而可能指导后续治疗起到提高患者预后水平的作用。但确切的提前时间还需进行前瞻性的研究或大量的回顾性研究来证实。

## 4 ctDNA MRD的检测指导早期NSCLC术后辅助治疗

据统计，接受手术的NSCLC患者的5年OS从Ⅰa1期的92%到Ⅲb期的26%^[30]^。所以即使是在早期疾病，也产生了临床上对辅助治疗的需求，以根除MRD。从2021年中国临床肿瘤学会（Chinese Society Of Clinical Oncology, CSCO）^[31]^和2021年美国国家综合癌症网络（National Comprehensive Cancer Network, NCCN）第1版^[32]^发布的《非小细胞肺癌诊疗指南》对比，可以看出针对早期NSCLC行根治性手术后辅助治疗方法的不同。

在CSCO指南中，Ⅰb期NSCLC（包括有高危因素的）因为缺乏高级别证据支持，一般不推荐辅助化疗，Ⅱa期患者根治术后可考虑含铂双药方案辅助化疗（2B类证据）。而NCCN指南中表示Ⅰb期-Ⅱa期NSCLC根治术后建议观察或高危人群进行化疗，Ⅱb期-Ⅲa期行化疗，对表皮生长因子受体（epidermal growth factor receptor, *EGFR*）突变阳性的Ⅰb期-Ⅲa期患者根治术后无论是否接受辅助化疗，均可以使用奥希替尼靶向辅助治疗。高危因素包括：①低分化肿瘤[包括肺神经内分泌肿瘤（不包括分化良好的神经内分泌肿瘤）]；②血管侵犯；③楔形切除；④肿瘤 > 4 cm；⑤内脏胸膜受累和淋巴结状态不明（Nx）。

综上两份指南存在的分歧，而在早期NSCLC根治术后的辅助治疗中，MRD的检测结果指导术后辅助治疗可产生显著的临床相关性。Chaudhuri等^[17]^用CAPP-seq检测ctDNA，在MRD出现36个月后，可检测到ctDNA的患者100%疾病进展，而未检测到ctDNA的患者93%（HR=43.4, *P* < 0.001）疾病无进展，MRD未检出ctDNA的患者远期生存率明显高于检测到ctDNA的患者（*P* < 0.001）。Chen等^[29]^发现术后3 d检测ctDNA阳性的患者，接受辅助治疗的RFS为269 d，未接受辅助治疗的RFS为111 d（*P*=0.018），ctDNA的变化与治疗疗效相关，且术后3 d检测ctDNA与DFS相关。在Kuang^[33]^对于术后ctDNA阳性的患者研究中，与化疗后ctDNA清零患者相比，化疗后ctDNA阳性患者的RFS较差（HR=8.68, *P*=0.022）。对于术后ctDNA阴性的患者，化疗后ctDNA阴性与化疗后ctDNA阳性患者相比有更好的远期疗效（HR=4.76, *P*=0.047）。该研究表明，化疗后ctDNA状态与RFS密切相关，有可能指导术后强化治疗。

综上所述，治疗后ctDNA可以由阳性转阴性，意味着手术或辅助治疗方案可以清除MRD，从而改变其疾病进展和生存。而ctDNA继续呈阳性或者是由阴转阳，可能预示着存在MRD，这可能是一个提示改变治疗方案的信号（表 1）。

## 5 总结

目前以ctDNA作为MRD的指标最常见，基于ctDNA提示MRD的研究在肠癌、乳腺癌、肌层浸润性尿路上皮癌及肺癌等术后患者的队列中，都显示出对肿瘤复发及转移预测的明显优势。现各类方法学在MRD场景孰优孰劣还值得进一步细分和探索，而NSCLC本身的分子克隆行为并不均一，从可能影响MRD监控效能的肿瘤基因变异数量考虑，驱动变异与非驱动变异的肿瘤，肿瘤突变负荷（tumor mutational burden, TMB）有极大的差异。一般来说，携带驱动变异的患者整体肿瘤基因变异远远少于并不携带驱动变异的患者，对于这两类人群我们应该如何去选择合适的MRD检测技术需进一步论证。MRD检测能够方便、精准、高效是支持它在临床应用的基础，而且ctDNA能比NSCLC治疗后的影像监测更早的预示着疾病的复发，给及时调整治疗方案提供了宝贵的时间。在早期NSCLC根治术后辅助治疗研究中，对合适的治疗方式存疑，而MRD可以作为一个理想的预测因素，对早期NSCLC根治术后患者进行精准的指导，从而提高患者的存活率和为改善预后作出贡献。此结论仍需大型前瞻性、随机对照临床研究对此进行深一步论证。

## References

[1] Sung H, Ferlay J, Siegel RL (2021). Global cancer statistics 2020: GLOBOCAN estimates of incidence and mortality worldwide for 36 cancers in 185 countries. CA Cancer J Clin.

[2] Reck M, Rabe KF (2017). Precision diagnosis and treatment for advanced non-small-cell lung cancer. N Engl J Med.

[3] Aberle DR, Adams AM, Berg CD (2011). Reduced lung-cancer mortality with low-dose computed tomographic screening. N Engl J Med.

[4] Kazemifard N, Sadeghi A, Varaminian B (2019). Circulating tumor DNA applications in monitoring the treatment of metastatic colorectal cancer patients. Gastroenterol Hepatol Bed Bench.

[5] Amatu A, Schirripa M, Tosi F (2019). High circulating methylated DNA is a negative predictive and prognostic marker in metastatic colorectal cancer patients treated with regorafenib. Front Oncol.

[6] Chae YK, Oh MS (2019). Detection of minimal residual disease using ctDNA in lung cancer: Current evidence and future directions. J Thorac Oncol.

[7] Deng T, Tang J, Zhou L (2019). Effective targeted therapy based on dynamic monitoring of gene mutations in non-small cell lung cancer. Transl Lung Cancer Res.

[8] Xia S, Ye J, Chen Y (2019). Parallel serial assessment of somatic mutation and methylation profile from circulating tumor DNA predicts treatment response and impending disease progression in osimertinib-treated lung adenocarcinoma patients. Transl Lung Cancer Res.

[9] Buder A, Hochmair MJ, Setinek U (2020). *EGFR* mutation tracking predicts survival in advanced EGFR-mutated non-small cell lung cancer patients treated with osimertinib. Transl Lung Cancer Res.

[10] Chae YK, Davis AA, Agte S (2019). Clinical implications of circulating tumor DNA tumor mutational burden (ctDNA TMB) in non-small cell lung cancer. Oncologist.

[11] Nabet BY, Esfahani M S, Moding EJ (2020). Noninvasive early identification of therapeutic benefit from immune checkpoint inhibition. Cell.

[12] Wang Y, Xie S, He B (2019). Effect of *EGFR* gene polymorphism on efficacy of chemotherapy combined with targeted therapy for non-small cell lung cancer in Chinese patients. Am J Cancer Res.

[13] Garcia-Murillas I, Schiavon G, Weigelt B (2015). Mutation tracking in circulating tumor DNA predicts relapse in early breast cancer. Sci Transl Med.

[14] Tie J, Wang Y, Tomasetti C (2016). Circulating tumor DNA analysis detects minimal residual disease and predicts recurrence in patients with stage Ⅱ colon cancer. Sci Transl Med.

[15] Powles T, Assaf ZJ, Davarpanah N (2021). ctDNA guiding adjuvant immunotherapy in urothelial carcinoma. Nature.

[16] Abbosh C, Birkbak NJ, Wilson GA (2017). Phylogenetic ctDNA analysis depicts early-stage lung cancer evolution. Nature.

[17] Chaudhuri AA, Chabon JJ, Lovejoy AF (2017). Early detection of molecular residual disease in localized lung cancer by circulating tumor DNA profiling. Cancer Discov.

[18] Newman AM, Bratman SV, To J (2014). An ultrasensitive method for quantitating circulating tumor DNA with broad patient coverage. Nat Med.

[19] Abbosh C, Birkbak NJ, Swanton C (2018). Early stage NSCLC - challenges to implementing ctDNA-based screening and MRD detection. Nat Rev Clin Oncol.

[20] Zhu G, Ye X, Dong Z (2015). Highly sensitive droplet digital PCR method for detection of *EGFR*-activating mutations in plasma cell-free DNA from patients with advanced non-small cell lung cancer. J Mol Diagn.

[21] Watanabe M, Kawaguchi T, Isa S (2015). Ultra-sensitive detection of the pretreatment *EGFR* T790M mutation in non-small cell lung cancer patients with an *EGFR*-activating mutation using droplet digital PCR. Clin Cancer Res.

[22] Vendrell JA, Mazieres J, Senal R (2019). Ultra-sensitive EGFR (T790M) detection as an independent prognostic marker for lung cancer patients harboring *EGFR* (del19) mutations and treated with first-generation TKIs. Clin Cancer Res.

[23] Chabon JJ, Hamilton EG, Kurtz DM (2020). Integrating genomic features for non-invasive early lung cancer detection. Nature.

[24] Zviran A, Schulman RC, Shah M (2020). Genome-wide cell-free DNA mutational integration enables ultra-sensitive cancer monitoring. Nat Med.

[25] Ettinger DS, Wood DE, Aisner DL (2017). Non-small cell lung cancer, version 5.2017, NCCN Clinical Practice Guidelines in Oncology. J Natl Compr Canc Netw.

[26] Kalemkerian GP, Akerley W, Bogner P (2013). Small cell lung cancer. J Natl Compr Canc Netw.

[27] Peng M, Huang Q, Yin W (2020). Circulating tumor DNA as a prognostic biomarker in localized non-small cell lung cancer. Front Oncol.

[28] Ohara S, Suda K, Sakai K (2020). Prognostic implications of preoperative versus postoperative circulating tumor DNA in surgically resected lung cancer patients: a pilot study. Transl Lung Cancer Res.

[29] Chen K, Zhao H, Shi Y (2019). Perioperative dynamic changes in circulating tumor DNA in patients with lung cancer (DYNAMIC). Clin Cancer Res.

[30] Goldstraw P, Chansky K, Crowley J (2016). The IASLC lung cancer staging project: Proposals for revision of the TNM stage groupings in the forthcoming (Eighth) edition of the TNM classification for lung cancer. J Thorac Oncol.

[b31] 31Guidelines of Chinese Society of Clinical Oncoligy (CSCO) Non-small cell lung cancer, 2021. Beijing: People's Medical Publishing House, 2021.中国临床肿瘤学会(CSCO)非小细胞肺癌诊疗指南, 2021. 北京: 人民卫生出版社, 2021.

[b32] 32NCCN clinical practice guidelines in oncology: non-small cell lung cancer (2021 version 1). (2020-11-25). URL: https://www.nccn.org/professionals/physician_gls/.

[33] Kuang PP, Li N, Liu Z (2020). Circulating tumor DNA analyses as a potential marker of recurrence and effectiveness of adjuvant chemotherapy for resected non-small-cell lung cancer. Front Oncol.

